# Polymorphism of HDAC9 Gene Is Associated with Increased Risk of Acute Coronary Syndrome in Chinese Han Population

**DOI:** 10.1155/2016/3746276

**Published:** 2016-08-24

**Authors:** Zhenhua Han, Xin Dong, Chaoying Zhang, Yue Wu, Zuyi Yuan, Xinhong Wang

**Affiliations:** ^1^Department of Cardiology Medicine, The Second Affiliated Hospital of Xi'an Jiaotong University, Xi'an, Shaanxi 710004, China; ^2^Department of Cardiovascular Medicine, First Affiliated Hospital of Medical College and Key Laboratory of Environment and Genes Related to Diseases, Xi'an Jiaotong University, Ministry of Education, Xi'an, Shaanxi, China

## Abstract

Recent genome-wide association studies (GWAS) have indicated an association of histone deacetylase 9 (HDAC9) genetic variant with large-vessel stroke and coronary artery disease, among the European population. However, whether HDAC9 gene is associated with an increased susceptibility to acute coronary syndrome (ACS) in Chinese Han population is not known. A total of 472 patients, including patients with ACS (*N* = 309), and those with chest pain syndrome (controls, *N* = 163) were enrolled. Genotyping for HDAC9 gene was performed using the ligation detection reaction assay. A series of statistical analyses were performed to investigate the correlation between HDAC9 gene SNPs and the susceptibility to ACS. The results revealed a significant association of rs2240419 with ACS risk in which the A allele (*P* = 0.047) and the A allele carriers (AA + AG) (*P* = 0.037) were more likely to be in ACS group as compared to those in the control group. None of two other SNPs, rs2389995 and rs2107595, were significantly associated with ACS risk (*P* > 0.05). Logistic regression analyses further revealed an increased risk for ACS in A allele carrier among rs2240419 genotypes, as compared to those with GG homozygotes (odds ratio: 1.869, 95% CI 1.143, 3.056,* P* = 0.013). A significant correlation between rs2240419 polymorphism of HDAC9 gene and the susceptibility to ACS in Chinese Han population was observed in this study.

## 1. Introduction

Acute coronary syndrome (ACS), which includes ST-segment elevation myocardial infarction (STEMI), non-ST-segment elevation myocardial infarction (NSTEMI), and unstable angina (UA), is a major cause of morbidity and mortality in the western world. The common denominator of ACS is a pathophysiologic process characterized by rupture of an atherosclerotic plaque, altered coronary vasomotor tone, platelet aggregation, and thrombosis. Compelling evidence from twin and epidemiological studies suggests a genetic basis underlying ACS [1, 2].

Histone deacetylases (HDACs) are a family of enzymes which balance the acetylating activities of histone acetyltransferases on chromatin remodeling and play essential roles in regulating gene transcription [3, 4]. About 18 mammalian HDACs have been identified and grouped into four classes based on their structural and biochemical characteristics [5]. HDAC9 is expressed in heart, T lymphocytes, endothelium, adipose tissues, vascular smooth muscle cells, and macrophages [5–7]. A recent genome-wide association study (GWAS) linked a variant of HDAC9 (rs11984041) on chromosome 7p21.1 with large-vessel ischemic stroke in individuals with European ancestry [8]. Han et al. reported that two SNPs, rs2389995 and rs2240419 on HDAC9, were significantly associated with large-vessel ischemic stroke risk in the Chinese population, although rs11984041 was not polymorphic in the Chinese population [9]. Upregulation of HDAC9 expression was observed in human atherosclerotic plaques including in carotid, aortic, and femoral plaques, which appears to implicate the altered HDAC9 expression in promoting atherosclerosis [6, 10]. Cao et al. showed that macrophage HDAC9 upregulation is atherogenic via suppression of cholesterol efflux and generation of alternatively activated macrophages in atherosclerosis [7]. Moreover, HDAC9 is also a common genetic variant that shares genetic susceptibility to ischemic stroke and coronary artery disease (CAD) [11].

In the present study, we aimed to examine the association between five SNPs of the HDAC9 gene and risk of ACS in a Chinese population. Our study represents one of the first efforts to associate risk alleles in HDAC9 gene region with ACS in the Chinese population.

## 2. Results

### 2.1. Clinical Characteristics

Baseline characteristics for all patients are shown in Table 2. Proportion of males, number of smokers, incidence of diabetes, heart rate, fasting blood glucose, and hs-CRP were higher in ACS group as compared to those in the control group. Left ventricular ejection fraction (LVEF) and ApoA1 level were significantly lower in patients with ACS as compared to those in the controls. No other significant intergroup differences were observed.

### 2.2. Association of Allele and Genotypic Frequency Distribution with Susceptibility of ACS

Two SNPs, rs11984041 and rs2023938, were found to be nonpolymorphic in the Chinese population because rare five-T allele carrier of SNP rs11984041 and five-G allele carrier of SNP rs2023938 were found among both patients with ACS and controls. Genotype distributions for other three SNPs were in Hardy-Weinberg equilibrium in both groups (*P* > 0.05, data not shown).  Table 3 shows the association of HDAC9 gene alleles or genotypes with ACS.

We compared allele and genotype frequencies of the three SNPs between the two groups. We observed an association of rs2240419 with ACS risk, in which the A allele (*P* = 0.047) and the A allele carriers (AA + AG) (*P* = 0.037) were more frequent in ACS group, in contrast to those in the control group, whereas, none of the two other SNPs, rs2389995 and rs2107595, were significantly associated with ACS risk (*P* > 0.05).

Multivariate logistic regression analysis of SNP rs2240419, adjusted for differences in age, sex, cigarette smoking, hypertension, diabetes, fasting blood glucose, hs-CRP, and lipoprotein variables, confirmed the independent association (Table 4). A allele carriers in rs2240419 genotypes had an increased ACS risk as compared to that associated with the GG homozygotes; the adjusted OR was 1.869 (95% CI 1.143, 3.056,* P* = 0.013). Diabetes, smoking, and hs-CRP were the other variables that independently contributed to the risk of ACS, the adjusted ORs being 2.306 (95% CI 1.232, 4.317,* P* = 0.009) for diabetes, 3.909 (95% CI 2.142, 7.134,* P* < 0.001) for smoking, and 1.126 (95% CI 1.043, 1.216,* P* = 0.002) for hs-CRP.

### 2.3. Relation between the rs2240419 Genotypes and Clinical Data

We also assessed the association of other clinical variables such as age, sex, cigarette smoking, hypertension, diabetes, fasting blood glucose, hs-CRP, and lipid profile, with rs2240419 genotypes (Table 5). No significant intergroup differences were observed among genetic frequencies in rs2240419 with these clinical parameters.

## 3. Discussion

In this study, we detected a significant correlation between rs2240419 polymorphism of HDAC9 gene and the susceptibility to ACS in a Chinese population, while the other four SNPs showed no such association.

A previous genome-wide association study (GWAS) demonstrated a significant association of SNP rs11984041 of HDAC9 gene with large-vessel stroke among populations of European ancestry [8]. Another GWAS on stroke in the European population revealed an association of polymorphic loci rs2107595 in HDAC9 gene susceptibility to large-vessel stroke [12]. Dichgans et al. evaluated the shared genetic determination of ischemic stroke and CAD and found that SNP rs2107595 on HDAC9 conferred susceptibility to both ischemic stroke and CAD among Caucasians [11]. Based on the HapMap data, SNP rs11984041 was found to be nonpolymorphic among the Chinese population. Our results are supported by two other studies which showed that SNP rs11984041 was not associated with stroke in a Chinese population, while two other SNPs, rs2389995 and rs2240419 of HDAC9, were significantly associated with large-vessel stroke risk [9, 13]. A recent study by Su et al. suggests that HDAC9 variant rs2107595 may not be associated with ischemic stroke risk in Chinese Han population [14]. Consistent with this study, we too found SNP rs11984041 to be nonpolymorphic in the Chinese population, and SNP rs2107595 was not significantly associated with ACS risk. This phenomenon may be attributed to genetic heterogeneity among different ethnic populations.

Eighteen HDACs are encoded by different genes and grouped into four classes on the basis of their structural and biochemical characteristics [15]. HDAC9 is a member of Class IIa HDACs. One of the best characterized mechanisms of action of HDAC9 is its ability to interact with MEF-2 and repress MEF-2 activity, which has been linked to suppression of cardiac hypertrophy [5, 16]. Moreover, HDAC9 expression was upregulated in human atherosclerotic plaques and was also a common genetic variant that shares the genetic susceptibility to ischemic stroke and coronary artery disease [11].

Cao et al. revealed the underlying molecular mechanisms of HDAC9 leading to increase of atherosclerosis in the in vivo models. HDAC9 deletion resulted in upregulation of lipid homeostatic genes, downregulation of inflammatory genes, and polarization of macrophages toward M2-like phenotype [7]. Recently another study found that HDAC9 regulates ox-LDL-induced endothelial cell apoptosis by participating in inflammatory reactions [17]. However, we did not observe a significant association between HDAC9 genetic variant and lipoproteins and inflammatory marker C-reactive protein. Thus, the mechanisms underlying the association of a mutant HDAC9 gene with ACS are still unknown.

## 4. Experimental Section

### 4.1. Subject Population

A total of 472 consecutive patients (309 patients with ACS and 163 patients with chest pain syndrome as controls) were admitted at the Department of Cardiology, the Second Affiliated Hospital of Medical College of Xi'an Jiaotong University, between January 2012 and January 2015. All patients underwent elective coronary angiography because of the clinical indication. Two independent interventional cardiologists analyzed the coronary angiography results. The diagnosis of ACS was based on angiography, in accordance with the ACC/AHA guidelines. Subjects with chest pain syndrome were defined as having atypical chest pain without significant angiographic coronary stenosis, aortic dissection, and pulmonary embolism. Exclusion criteria included cancer, advanced liver or renal dysfunction, active inflammatory or autoimmune diseases, and hematological diseases. The research protocol was approved by the Ethics Committee at the Second Affiliated Hospital of Xi'an Jiaotong University. Written informed consent was obtained from all the patients; the study complied with the principles enshrined in the Declaration of Helsinki. Demographic variables, cardiovascular risk factors, and past cardiovascular medical history were recorded for all patients.

### 4.2. Biochemical Analysis

Fasting blood glucose and serum concentrations of lipoproteins, apolipoproteins, and other biochemical parameters were measured in the clinical Biochemistry Department, at the Second Affiliated Hospital of the Medical College of Xi'an Jiaotong University.

### 4.3. DNA Extraction and Genotyping

Genomic DNA was extracted from peripheral blood lymphocytes (PBLs) using a commercial blood DNA extraction kit (Tiangen Biotech Co., Ltd., Beijing, China) following the manufacturer's protocol. Guided by the literature search, five SNPs in the HDAC9 gene variants were chosen for this study (SNP1 rs11984041, SNP2 rs2107595, SNP3 rs2023938, SNP4 rs2389995, and SNP5 rs2240419) [6, 8–11, 13, 14]. Genotyping for HDAC9 was performed using the ligation detection reaction (LDR) assay (Shanghai Biowing Applied Biotechnology Company) [18, 19]. All PCR primers and gene probes used in LDR were designed using Primer 3.0 and Oligo 6.0 software (listed in Table 1). Each set of LDR probes is comprised of one common probe and two discriminating probes for the two types. DNA sequences of the HDAC9 gene were amplified by a multiplex polymerase chain reaction (PCR) method. All PCR reactions were carried out in a final volume of 20 *µ*L containing 2 *µ*L PCR buffer (1x), 3.0 mmol/L MgCl_2_, 2.0 mmol/L dNTP, 2 *µ*L primer mix, 0.2 *µ*L Taq Polymerase, and 1 *µ*L genomic DNA (50 ng/*µ*L). The amplification procedure was performed with an initial denaturation at 95°C for 2 min, followed by 40 cycles of denaturation at 94°C for 30 s, annealing at 53°C for 90 s, and extension at 65°C for 30 s, followed by a final extension at 65°C for 10 min in Gene Amp PCR system 9600 (Norwalk, CT, USA). The LDRs were performed in a final volume of 10 *µ*L containing 1 *µ*L buffer (1x), 1 *µ*L probe mix (2 pmol/*µ*L), 0.05 *µ*L Taq DNA ligase (NEB Biotechnology), and 4 *µ*L of multi-PCR product. The LDR included an initial incubation at 95°C for 2 min, followed by 40 cycles at 94°C for 15 s and 50°C for 25 s. The LDR fluorescent products were analyzed by ABI sequencer 3730. The quality of genotyping was controlled using blinded blood duplicates.

### 4.4. Statistical Analysis

All statistical analyses were conducted using SPSS software package, version 18.0 (SPSS Inc., Chicago, IL, USA). Quantitative variables are expressed as means ± Standard Error (SE) and categorical variables as frequency and percentage. Intergroup differences were assessed by Chi-square test or* t*-test. The genotype distributions for HDAC9 SNPs were tested for Hardy-Weinberg equilibrium. Owing to the small number of patients with AA homozygote in rs2240419 (*N* = 18 and *N* = 9 for ACS and control, resp.), AA and AG patients in rs2240419 were combined into a single A allele group (AA + AG) to improve the statistical power. The odds ratio (OR) and 95% confidence interval (95% CI) were calculated using binary logistic regression model to access associations between SNP rs2240419 and ACS risk. A 2-tailed* P* < 0.05 was considered statistically significant.

## 5. Conclusion

In this study, we demonstrated an association between rs2240419 polymorphism and risk of ACS in a Chinese population. Our findings also attest to the genetic heterogeneity between Chinese and other ethnic groups. However, several potential limitations of our study should be noted. Samples collected from a single center may not be sufficient and the chosen SNPs may not represent the entire HDAC9 gene. Future studies with larger sample size representative of the Chinese population are required to validate our findings.

## Figures and Tables

**Table 1:** Primers and probes sequences (5′-3′) of HDAC9 SNPs for PCR and LDR.

Primers and probes	Sequences (5′-3′)		Length
PCR primers			
rs11984041	TTACTAGATCTGTCATGCAC	TGTGGTGGATGCACTGTATG	105 bp
rs2389995	ACCCCAGAGAGAGTCCTCTA	CTGGATTAAAGAGTGCCAAC	94 bp
rs2107595	GAAGGATGAGGAGCCATTAC	TTTGTGTGCTTGTACATTC	89 bp
rs2023938	TCATTTCAACGGCTTCACAG	TGAGGGCCTTGACTATTTAG	118 bp
rs2240419	CCACAGCTAAGAAGTATCAC	CCATGTTATGAAAAGCAGCC	101 bp
Probes			
rs11984041_modify	P-TGGCATCTCCATACAGTGCATCCACTTTTTTTTTTTTTTTTTTTTTTTTTTTTTTTTTTTTTTTTTTTTTTTTTTTTTT-FAM		
rs11984041_C	TTTTTTTTTTTTTTTTTTTTTTTTTTTTTTTTTTTTTTTTTTTTTTTTTTTTGAAGCTGGTAACTTCTACG		150 bp
rs11984041_T	TTTTTTTTTTTTTTTTTTTTTTTTTTTTTTTTTTTTTTTTTTTTTTTTTTTTTTGAAGCTGGTAACTTCTACA		152 bp
rs2389995_modify	P-GCCTTCTAAGGATCCAAGGAGAAGTTTTTTTTTTTTTTTTTTTTTTTTTTTTTTTTTTTTTTTTTTTTTTTTTTTTTTT-FAM		
rs2389995_A	TTTTTTTTTTTTTTTTTTTTTTTTTTTTTTTTTTTTTTTTTTTTTTTTTTAGAGAGAGTCCTCTAGCTCTCTTGTT		155 bp
rs2389995_G	TTTTTTTTTTTTTTTTTTTTTTTTTTTTTTTTTTTTTTTTTTTTTTTTTTTTAGAGAGAGTCCTCTAGCTCTCTTGTC		157 bp
rs2107595_modify	P-CAAAAAAGAATGTACAAGCACACAATTTTTTTTTTTTTTTTTTTTTTTTTTTTTTTTTTTTTTTTTTTTTTTTTTTTTT-FAM		
rs2107595_C	TTTTTTTTTTTTTTTTTTTTTTTTTTTTTTTTTTTTTTTTTTTTTTTTTTTTTTTTACTGTGGGACAAAAACATTTTCCCG		160 bp
rs2107595_T	TTTTTTTTTTTTTTTTTTTTTTTTTTTTTTTTTTTTTTTTTTTTTTTTTTTTTTTTTTACTGTGGGACAAAAACATTTTCCCA		162 bp
rs2023938_modify	P-TGCATTTTATTAACTGTGAAGCCGTTTTTTTTTTTTTTTTTTTTTTTTTTTTTTTTTTTTTTTTTTTTTTTTTTTTTTT-FAM		
rs2023938_A	TTTTTTTTTTTTTTTTTTTTTTTTTTTTTTTTTTTTTTTTTTTTTTTTTTTTTTTTTTTTAGGTTAACACAGTTGTTTTGTCTGAT		165 bp
rs2023938_G	TTTTTTTTTTTTTTTTTTTTTTTTTTTTTTTTTTTTTTTTTTTTTTTTTTTTTTTTTTTTTTAGGTTAACACAGTTGTTTTGTCTGAC		167 bp
rs2240419_modify	P-AGATTGGTGCCAGTCTTGGTTGTGATTTTTTTTTTTTTTTTTTTTTTTTTTTTTTTTTTTTTTTTTTTTTTTTTTTTTT-FAM		
rs2240419_A	TTTTTTTTTTTTTTTTTTTTTTTTTTTTTTTTTTTTTTTTTTTTTTTTTTTTTTTTTTTTTTTTTGCAGCCCTGTGCACTGGTGAAGAGCT		170 bp
rs2240419_G	TTTTTTTTTTTTTTTTTTTTTTTTTTTTTTTTTTTTTTTTTTTTTTTTTTTTTTTTTTTTTTTTTTTGCAGCCCTGTGCACTGGTGAAGAGCC		172 bp

SNP, single nucleotide polymorphism; PCR, polymerase chain reaction; LDR, ligation detection reaction.

**Table 2:** Clinical characteristics of subjects by study group.

Characteristics	Control (*N* = 163)	ACS (*N* = 309)	*P*
Sex (m/f)	94/69	229/80	<0.001
Age (years)	59.66 ± 10.82	60.61 ± 11.98	0.401
Hypertension (%)	54.6	57.9	0.488
Diabetes (%)	13.1	25.2	0.002
Smoking (%)	29.4	55	<0.001
SBP (mmHg)	127.54 ± 22.74	127.74 ± 22.84	0.927
DBP (mmHg)	78.82 ± 13.55	78.83 ± 13.89	0.998
HR (bpm)	71.60 ± 14.64	74.72 ± 14.56	0.028
LVEF (%)	63.45 ± 12.19	59.05 ± 12.16	<0.001
hs-CRP (mg/L)	2.23 ± 3.07	4.34 ± 4.26	<0.001
FBS (mmol/L)	5.88 ± 1.98	7.10 ± 3.43	<0.001
TC (mmol/L)	3.90 ± 0.91	3.85 ± 0.96	0.556
TG (mmol/L)	1.60 ± 1.21	1.72 ± 1.18	0.302
HDL-C (mmol/L)	0.98 ± 0.24	0.94 ± 0.63	0.484
LDL-C (mmol/L)	2.32 ± 0.78	2.28 ± 0.85	0.617
ApoA1 (g/L)	1.14 ± 0.21	1.07 ± 0.19	<0.001
ApoB (g/L)	0.76 ± 0.21	0.77 ± 0.22	0.752
Statins (%)	83.4	98.0	<0.001
ACEI/ARB (%)	69.9	88.2	<0.001
*β*-blocker (%)	62.0	86.6	<0.001
CCB (%)	24.5	20.3	0.293

ACS, acute coronary syndrome; SBP, systolic blood pressure; DBP, diastolic blood pressure; HR, heart rate; LVEF, left ventricular ejection fraction; hs-CRP, high sensitivity C-reactive protein; FBS, fasting blood sugar; TC, total cholesterol; TG, triglycerides; HDL-C, high-density lipoprotein cholesterol; LDL-C, low-density lipoprotein cholesterol; ApoA1, apolipoprotein A1; ApoB, apolipoprotein B; ACEI, angiotensin converting enzyme inhibition; ARB, angiotensin receptor blocker; CCB, calcium channel blocker.

**Table 3:** Genotypes and alleles distribution of HDAC9 gene SNPs in ACS and control.

	Control		ACS		*P*
	*N*	%	*N*	%	
rs2389995					
Genotypes					
AA	113	69.30	198	64.10	0.377
AG	45	27.60	104	33.70	
GG	5	3.10	7	2.30	
Allele					
A	271	83.10	500	80.90	0.401
G	55	16.90	118	19.10	
rs2107595					
Genotypes					
CC	78	47.90	139	45.00	0.183
CT	73	44.80	130	42.10	
TT	12	7.40	40	12.90	
Allele					
C	229	70.20	408	66.00	0.188
T	97	29.80	210	34.00	
rs2240419					
Genotypes					
AA	9	5.50	18	5.80	0.037^*∗*^
AG	48	29.40	127	41.10	
GG	106	65.00	164	53.10	
Allele					
A	66	20.20	163	26.40	0.047^*∗*^
G	260	79.80	455	73.60	

ACS, acute coronary syndrome; *∗* denotes *P* < 0.05.

**Table 4:** Results of logistic regression analysis showing risk factors for ACS.

Covariates	Odds ratio (95% CI)	*P*
rs2240419	1.869 (1.143, 3.056)	0.013
Sex	0.923 (0.516, 1.653)	0.788
Age	1.018 (0.996, 1.040)	0.112
Hypertension	1.334 (0.827, 2.152)	0.238
Diabetes	2.306 (1.232, 4.317)	0.009
Smoking	3.909 (2.142, 7.134)	<0.001
TC	1.646 (0.529, 5.124)	0.390
TG	1.112 (0.886, 1.395)	0.360
HDL-C	1.432 (0.556, 3.690)	0.457
LDL-C	0.753 (0.226, 2.507)	0.644
ApoA1 (g/L)	0.267 (0.035, 2.037)	0.203
ApoB (g/L)	0.435 (0.028, 6.742)	0.552
hs-CRP	1.126 (1.043, 1.216)	0.002

ACS, acute coronary syndrome; TC, total cholesterol; TG, triglycerides; HDL-C, high-density lipoprotein cholesterol; LDL-C, low-density lipoprotein cholesterol; ApoA1, apolipoprotein A1; ApoB, apolipoprotein B.

**Table 5:** Association of rs2240419 genotypes and clinical characteristics.

Covariates	AA + AG	GG	*P*
Sex (m/f)	136/66	187/83	0.655
Age	59.30 ± 11.40	61.01 ± 11.70	0.111
Hypertension	55.9	57.4	0.750
Diabetes	25.0	18.0	0.068
Smoking	45.0	47.0	0.668
TC	3.82 ± 0.88	3.90 ± 0.98	0.356
TG	1.64 ± 1.01	1.71 ± 1.32	0.497
HDL-C	0.91 ± 0.23	0.99 ± 0.67	0.158
LDL-C	2.26 ± 0.79	2.32 ± 0.85	0.469
ApoA1	1.08 ± 0.20	1.10 ± 0.21	0.354
ApoB	0.75 ± 0.21	0.77 ± 0.22	0.424
hs-CRP	4.03 ± 4.40	3.33 ± 3.72	0.091

TC, total cholesterol; TG, triglycerides; HDL-C, high-density lipoprotein cholesterol; LDL-C, low-density lipoprotein cholesterol; ApoA1, apolipoprotein A1; ApoB, apolipoprotein B.
